# Situational assessment and epidemiology of HIV, HBV and HCV among people who use and inject drugs in Ghana

**DOI:** 10.1371/journal.pone.0305923

**Published:** 2024-08-26

**Authors:** Chris Guure, Samuel Dery, Carlota Baptista da Silva, Comfort Asamoah-Adu, Stephen Ayisi-Addo, Kofi Diaba, Maria-Goretti Loglo, Adamu Mohammed, Yaw Akrasi Sarpong, Samuel Hanu, Kwasi Torpey

**Affiliations:** 1 Department of Biostatistics, School of Public Health, University of Ghana, Legon-Accra, Ghana; 2 International Consultant, Harm Reduction and Key population Expert, Lisbon, Portugal; 3 West Africa Program to Combat AIDS, Accra, Ghana; 4 National AIDS/STI Control Programme, Ghana Health Service, Accra, Ghana; 5 International Drug Policy Consortium, East Legon, Accra, Ghana; 6 West Africa Behavioural Health Addictions and Recovery Management, Accra, Ghana; 7 West Africa Drug Policy Network (WADPN)-Ghana Chapter, Accra, Ghana; 8 Harm Reduction Alliance Ghana, Accra, Ghana; 9 Department of Population Family and Reproductive Health, School of Public Health, University of Ghana, Legon-Accra, Ghana; University of Cincinnati College of Medicine, UNITED STATES

## Abstract

**Introduction:**

People who inject drugs (PWID) and people who use drugs (PWUD) are an important population group that remain under-served in Ghana. Though PWID and PWUD are among the key populations most-at-risk to acquire sexually transmitted or blood-borne diseases, they are among those with the least access to human immunodeficiency (HIV), hepatitis B (HBV) and hepatitis C (HCV) viruses’ prevention, care and treatment services in Ghana due to lack of data on them. We provide a rapid assessment of the PWUD and PWID situation in Ghana.

**Methods:**

This rapid cross-sectional design undertook consultative meetings between the study team and relevant stakeholders, including Civil Society Organizations (CSO) working with PWUD/PWID. The assessment considered a representative sample of PWID and PWUD. It was conducted in four (4) selected regions of Ghana (Greater Accra, Ashanti, Western, and Northern). Overall, 323 participants were interviewed using respondent-driven sampling (RDS) approach. Information obtained from participants were demographics, HIV risk behaviors, human immunodeficiency (HIV) and sexually transmitted infections (STI)-related knowledge HIV/HCV/HBV screening, attitude, and practices among others. Analyses were conducted using Stata version 17 and RDSAT version 7.1.46 software.

**Findings:**

Drug use was found to be more prevalent among the youth with a median age of 37 years. Majority of the respondents were males (86%). About 28% of the female respondents identified themselves as sex workers, while about 74% have been involved in transactional sex. The median age at which respondents started using and injecting drugs was 20 and 22 years respectively. Majority (68%) of the respondents consume drugs through smoking, with 20% through snorting, inhaling or swallowing and 12% through injection. The drug mostly used among the respondents was heroin (52%). The most commonly injected drug was cocaine (55%). About 64.7% of respondents reported mixing two or more drugs. HIV prevalence among respondents was 2.5%, 12.3% among women and 17.7% among women engaged in sex work, highlighting the overlap vulnerability. The prevalence of hepatitis C was 6.0%, and Hepatitis B was 4.5%. Access to care is limited, with 63% of the respondents never been tested for HIV.

**Conclusion:**

These rapid assessment findings reveal the challenging conditions for people who use and inject drugs coupled with a relatively high prevalence of HIV and Hepatitis C compared to the general population. However, it also reveals that Ghana has a window of opportunity to prevent an exponential spread of HIV and Hepatitis in this population. Therefore, there is the need to implement prevention and treatment programs for HIV and hepatitis among people who use and inject drugs including essential strategies for an enabling environment in Ghana.

## Introduction

People who inject drugs (PWID) and people who use drugs (PWUD) are an important population group that remain under-served. They are among the key populations most at risk to acquire or transmit HIV or other sexually transmitted or blood-borne infections. Yet they are also among those with the least access to HIV prevention, care and treatment services because their drug use is often stigmatized and criminalized. It is important to recognize their vulnerability and take concrete steps to provide appropriate services that address their needs. The number of drug users in Africa is projected to rise in the next decade by as much as 40 percent [1]. Although a rise in people who use drugs is predicted to go up by 11% globally, it is likely to be particularly pronounced in Africa because of the relatively young population.

There is limited published information about people who use and inject drugs in sub-Saharan Africa, though HIV is an important risk for this population and one of the leading causes of deaths and disabilities [2]. The Global Burden of Disease Study 2017 estimated that there were 585,000 deaths and 42 million years of “healthy” life lost due to drug use [3]. Males were estimated to constitute 72% of the deaths. More than half of the deaths (52 percent) in 2017 were attributable to untreated hepatitis C, which led to liver cancer and cirrhosis [3].

Additionally, about 29 percent were because of drug use disorders, mainly from the use of opioids (66 percent of deaths from drug use disorders) and 11 percent due to HIV and AIDS, this was according to the World Drug Report 2019, [4]. The World Drug Report estimated the number of drug users as of 2019 at 275 million worldwide, up from 226 million in 2010. It is estimated that about 11.3 million inject drugs, with approximately 1.4 million infected with HIV, 5.6 million with hepatitis C, and almost 1.2 million living with both diseases [1]. According to the 2020 Global Harm Reduction report by Harm Reduction International, most countries in sub-Saharan Africa have poor collection and availability of data on drug use.

Furthermore, the health of people who use drugs, and harm reduction services for people who inject drugs are limited [5].^.^The number of people who inject drugs (PWID) is estimated to be between 560,000 and 2.7 million, a range that demonstrates the paucity of data. It is further estimated that between 23% and 39% of new HCV infections are attributable to people who inject drugs [4].

In Ghana, several reports mention an increase in domestic drug use. A study conducted in Kumasi, on multiple HIV vulnerabilities of men and women who inject drugs established that there is low knowledge of HIV transmission through drug use, reuse and sharing of injectable equipment among PWIDs [6]. It also found out that condom use with intimate partners who also inject drugs was low, rendering them vulnerable to HIV, blood-borne and other sexually transmitted infections [6]. Needle sharing and other high-risk behaviors are high among people who inject drugs [7]. Understanding the epidemiology of HIV transmission among PWID and PWUD will help prevent transmission within the community and the general population [8].

A study conducted among prison-inmates found prisoners who reported ever having injected drugs, had 5.7, 5.4, and 5.3 odds of testing positive for HIV, HBV, and HCV while those who reported sharing needles or injection implements, had 2.0, 1.9, and 1.9, odds of testing positive for HIV, HBV, and HCV respectively [9]. Additionally, Messersmith and colleagues investigated used syringe use and unsafe sex among people who inject drugs in Kumasi, Ghana. They found that of the used syringes tested for HIV, HCV and HBV, the prevalence estimates were 3% (HIV), 2% (HCV), and 9% (HBV) [10].

As a first step in designing HIV, Hepatitis and harm reduction interventions such as needle and syringe programs, (NSPs) and Opioid substitution therapy (OST) for PWID and PWUD in Ghana, it is critical to understand the dynamics of drugs injection and use, including barriers to assessing services, policies and knowledge gaps that would influence healthcare-seeking behavior of persons who use or inject drugs in Ghana. The aim of this study was to describe the population of PWID and PWUD, estimate the prevalence of HIV, HBV and HCV and establish whether there is a statistically significant difference in disease among this population by sex.

## Methods

### Overall approach to the research study

The study team approached this work through an overarching principle of all-inclusiveness and collaboration. The team constituted a Community Advisory Group (CAG) that comprised of active/former people who use and/or inject drugs. Others were stakeholders engaged directly or indirectly in harm reduction services such as, policies, programs and practices that aim to minimize negative health, social and legal impacts associated with drug use and injection, grounded in justice and human rights, without judgment, coercion, discrimination, or requiring that people stop using drugs as a precondition for support. The role of the CAG included advising the study team in the planning, selection of geographical areas, identification of PWUD/PWID, field supervision, validation of findings and challenges/safety concerns. This approach is in line with “nothing for us without us”.

### Stakeholders’ and consultative meetings

Stakeholders’ consultative meetings were organized between the study team, relevant stakeholders and the technical leadership across the selected regions. Civil Society Organizations (CSOs) and Non-Governmental Organizations (NGOs) working with PWUD/PWID were engaged in the consultations. The team undertook a rapid stakeholders’ power mapping to determine who is doing what in terms of service provision and their position towards harm reduction (includes health and non-health institutions/actors working on harm reduction, rehabilitation/detoxification, like the Narcotics Control Commission, Ghana Health Service, Ghana Police Service, CSOs and other implementing partners). During these consultative meetings, decisions were made on which communities in each of the selected administrative regions should be included in the rapid assessment. These discussions enabled the investigators to purposively select communities within regions representing Ghana’s three ecological zones.

### Study design and geographic coverage/study sites

The study was a cross-sectional evaluation and viral infections among PWID and PWUD population in Ghana using an RDS sampling approach. This was to ensure adequate representation of the diverse categories. RDS uses a non-probability sampling approach (snowball via purposive selection of seeds) and combines it with a mathematical modelling technique to weight the non-randomly selected sample in other to generate reliable estimates of the population parameters. PWUD/PWID 18 years and above who have used one of the following drugs (Heroin, Cocaine, Crack, Crystal Meth/Methamphetamine, other opioids such as tramadol, morphine and pethidine) in the past 6 months were included in the study. The survey started on the 11^th^ of November 2021 and ended on the 18^th^ of December 2021. All participants provided written informed consent. This study was conducted in four (4) selected regions of Ghana: Greater Accra, Ashanti, Western, and Northern regions.

### Behavioral questionnaire

This study employed a quantitative approach to determine the prevalence of HIV, Hepatitis B & C among PWUD and PWID in Ghana. Furthermore, the structured individual participant questionnaire sought to elicit information on socio-demographic characteristics, extent and types of drugs used, risky sexual behaviors, access to services, HIV/STI-related knowledge, attitudes, and practice as well as injection and non-injection drug use. It also assessed social issues including educational, health and population specific issues among PWID and PWUD.

### Sample size determination

The modified Cochran’s formula that incorporates the design effect, stratification and non-response rate at the individual level as provided below was used to obtain sample size for the study:

$$n=Strata\times \{{Z^{2}}_{1-\alpha /2}(p)(1-p)/{\left[e\right]}^{2}\}\times (r+1),$$

where *n* is the required sample size (number of study participants in the study), *Z*^2^_1−α/2_ = 1.96 is the standard normal variate at type I error (*α*) = 5%. The anticipated proportion of study participants who use drugs and are HIV positive was *p* = 5.2% according to a recent study on the prevalence and risk factors associated with HIV and tuberculosis in people who use drugs in Abidjan, Ivory Coast with a margin of error of *e* = 5% [11]. Substituting the parameters into the above expression, the total number of participants required for the study was 303.

### Sampling approach (Respondent Driven Sampling)

Respondent-driven sampling (RDS) was used to overcome the difficulties in enrolling the PWUD and PWID since it is a hard-to-reach population. The RDS was chosen for this study because it reduces the biases inherent in referral methods by controlling for the number of coupons and making statistical adjustments that attempt to account for personal network size and similarity among persons within the same social networks. Using the RDS approach, seeds were selected—Seeds were identified with the help of key informants and the Community Advisory Group. This RDS method is based on recognizing that peers with respect to hard-to-reach populations are better placed to contact the population than the usual field staff or research assistants. The implementation of RDS is premised on the selection of a “seed” at the initial stages based on pre-existing contacts with a person or people within the population network. The initial seeds (were selected by stakeholders) were interviewed and formed wave zero (0) of the sample selection. In this study, four (4) seeds were selected in each of the four regions that were involved in the study. Seeds were selected to include PWUD, PWID and female respondents. These seeds were given a set number of coded recruitment coupons, and they were requested to give these to persons in their social circle who inject or use drugs. This recruitment approach then turned into “waves” whereby the first set of participants referred to the study staff by the seeds (first recruits) then became wave one (1) (second recruits) and formed the second set of respondents to be interviewed. Wave two (2) constituted respondents who were also referred to the study staff by the wave one (1) respondents. Each cycle of recruitment and participation added an additional sampling wave and effectively created recruitment chains. The number of recruits per person was restricted to three (3) to facilitate long chains, which may help recruit diverse social networks. All those who were referred and came to the survey site were screened for eligibility. They were enrolled if they met the survey inclusion criteria and consented to participate. All interviews were conducted in selected venues or offices which were considered very safe and conducive for the study. All data collectors were trained in data collection procedures.

### Laboratory methods

#### Biological sampling

Venous blood was collected using vacuum tubes containing the anti-coagulant ethylene-diamine-tetra-acetic acid (EDTA). *HIV* testing was conducted on site with 6ml of whole blood using First Response HIV 1+2 / Syphilis Combo Card Test. It is a rapid, qualitative screening, in vitro diagnostic test for the detection of antibodies specific to HIV (type 1 & 2) and *Treponema pallidum*. Reactive specimens for HIV was confirmed further with Oraquick test kits. A third HIV test using SD Bioline was employed. These three reactive sequential tests were used to declare a participant as HIV positive if the individual was reactive to all. All clients were counseled and those willing to take their results received them immediately. Clients whose results were positive for HIV, were linked up with a local NGO participating in the study for further counseling and referred to an appropriate treatment facility (where the study counsellor works). *Hepatitis B* was tested using First Response HBsAg card test (Premier Medical Corporation). This is a chromatographic immunoassay for qualitative detection of Hepatitis B Surface Antigen in whole blood, plasma or serum. *Hepatitis C* was tested using First Response^®^ HCV Card Test. This is a chromatographic immunoassay for the qualitative detection of the antibodies against hepatitis C virus (HCV Ab) in human serum, plasma, or whole blood (venous & capillary blood) specimens.

#### Biological samples transport to UG-NMIMR

After blood sample collection, the laboratory staff oversaw sample processing, storage and transportation to UG-NMIMR for HIV-1 viral load measurements among respondents with positive HIV antibody test results. Study specific procedures for the transport (including cold chain requirements and chain of study) and management of study specimens were followed to ensure the integrity of specimens. When study was completed at each site, the blood samples were transported to the UG-NMIMR in cool boxes with ice packs. At the close of each day, all samples were transported to a designated health facility laboratory. Staff involved in all activities were trained in the correct procedures including how to pack and transfer samples and maintain cold chain.

### Data management and analysis

All quantitative data from the individual participants were captured using Research Electronic Data Capture (REDCap) mobile application software. All electronic data capturing devices were password-protected. Analysis of the quantitative data was done descriptively and presented in the form of medians, proportions (percentages), bar and pie charts. These analyses were preceded by data cleaning, recoding of relevant variables. In other to obtain unbiased estimates, we run diagnostic procedures by taking into consideration homophily and convergence analyses to ensure that the data met the assumptions of RDS. We obtained prevalence estimates and 95% confidence intervals via bootstrapped approach using the enhanced data smoothing estimator in the RDSAT 7.1 (Cornell University, Ithaca, NY) software. In the RDSAT software, we specified 15,000 bootstrap re-samples with the algorithm type set as “enhanced data-smoothing”. The RDSAT was used to perform key group and trait correspondence, recruitment, transition probabilities, demographically adjusted recruitment matrices. The software was used to calculate sample weights (based on the sample of interest–HIV status), which were used to obtain adjusted estimates in the analyses.

### Ethics declarations

#### Ethics approval and consent to participate

The study received ethics approval from the University of Ghana College of Health Sciences Institutional Review Board (CHS-Et/M.6 –p4.8/2020-2021)–All data presented are from people who provided written informed consent to participate in the study.

## Results

### Characteristics of study participants

The study interviewed a total of 323 respondents from the four regions. Most of the respondents were from the Greater Accra region (41.5%), and the least (6.1%) from the Northern region, Table 1. The median age of the respondents was 37 (IQR: 28, 44) years. Most (39.7%) of the respondents had only attained junior high or middle school education. Most (38.6%) of the respondents were unemployed, and majority (57.0%) were single, but a third (34.1%) were living with sexual partners. A fifth (20.3%) of the respondents earned less than GHc 200.00 ($35.00) per month and 6.5% earned above GHc 2000.00 ($344.00) per month. About 3.9% of the respondents were sex workers and 0.7%, men who have sex with men. The median age at which respondents started drug use and injection were 20 (IQR: 18, 26) years and 22 (IQR: 18, 30) years respectively, Table 1.

**Table 1:** Characteristics of respondents who use drugs and according to sex.

		Gender	
	Total	Males	Females
N	323	278	45
Factor	%	%	%
**Age of participant, Median (IQR)**	37 (28, 44)	37 (31, 44)	26 (23, 37)
**Age group of participants**			
** **19–29 years	27.7	22.1	62.1
** **30–39 years	35.1	36.9	23.7
** **40–49 years	22.1	24.3	8.7
** **>49 years	14.6	16.1	5.5
** **Non-response	0.5	0.6	0.0
**Highest level of education**			
** **Never attended school	2.3	1.9	4.8
** **Primary	24.9	24.6	26.9
** **JHS/middle school	39.7	39.9	38.2
** **SHS/SSS/Voc./Tech.	29.7	30.3	26.2
** **Tertiary	3.4	3.3	3.9
**Employment status**			
** **Employed full-time	36.0	37.5	26.5
** **Employed part-time	24.1	26.6	8.3
** **Full-time student	0.6	0.3	2.6
** **Retired	0.7	0.1	4.3
** **Unemployed	38.6	35.5	58.3
**Marital status**			
** **Single, never married	57.0	55.0	69.9
** **Married	27.4	30.2	9.9
** **Separated/divorced	7.5	8.2	3.3
** **Widowed	8.0	6.6	16.8
**Currently living with sexual partner**			
** **Yes	34.1	28.9	66.4
** **No	65.9	71.1	33.6
**Monthly income**			
** **Less than 200 GHC	20.3	18.5	31.5
** **200 to 500 GHC	23.8	22.4	32.4
** **500 to 1000 GHC	26.0	29.2	6.1
** **1000 to 2000 GHC	18.7	20.8	5.8
** **Above 2000 GHC	6.5	5.7	11.2
** **Don’t know	2.5	1.2	10.8
** **Refuse to answer	2.2	2.2	2.1
**How respondents identify self**			
SW	3.9	0.0	28.0
MSM	0.7	0.8	0.0
General population	94.4	98	72.0
Refuse to answer	1.1	1.2	0.0
**Age at start of using of drugs, Median (IQR)**	20 (18, 26)	21 (18, 27)	20 (18, 24)
**Age group at start of using drugs**			
** **<15 years	4.4	4.3	5.4
** **15–19 years	31.1	29.2	42.6
** **20–24 years	25.8	23.9	37.6
** **25–29 years	12.0	12.9	6.2
** **>30 years	16.9	18.3	8.3
** **Non-response	9.7	11.3	0.0
**Age at start of injecting drugs, Median (IQR)**	22 (18, 30)	22 (19, 30)	16 (16, 16)
**Age group at start of injecting drugs**			
** **<15 years	5.6	5.8	0.0
** **15–19 years	23.9	21.9	100
** **20–24 years	25.0	25.6	0.0
** **25–29 years	16.6	17.1	0.0
** **>30 years	28.9	29.7	0.0
**Regions**			
** **Greater Accra	41.5	40.4	48.6
** **Ashanti	23.4	23.3	24.1
** **Western	28.9	30.0	22.4
** **Northern	6.1	6.3	4.9
**Do you commonly use two or more drugs together?**			
** **Yes	64.7	67.3	48.3
** **No	25.8	21.6	51.7
** **Non-response	9.5	11.0	0.0
**Ever changed from one drug to another**			
** **Yes	74.2	75.6	65.1
** **No	19.1	16.6	34.9
** **Non-response	6.7	7.8	0.0
**Drug changed to**			
** **Cocaine	35.8	34.6	44.5
** **Crack	21.5	20.4	28.8
** **Heroin	29.1	30.9	16.5
** **Marijuana	3.9	4.2	2.0
** **Campucheas (heroin mixed with marijuana)	0.6	0.7	0.0
** **Cocktail (marijuana + heroin + crack)	1.2	1.3	0.0
** **Tramadol	5.2	5.7	1.7
** **Alcohol	0.8	0.9	0.0
** **Cigarette	0.4	0.3	1.0
** **Prescription drugs	1.5	0.9	5.6

*Polydrug use by sex*. About two-thirds (64.7%) of the respondents used two or more drugs together. The drugs mostly used among the respondents were crack (60.6%), heroin (52.9%), and marijuana (49.9%). The distribution of the drugs mostly used among respondents by sex is provided in Table 1.

**Fig 1:**
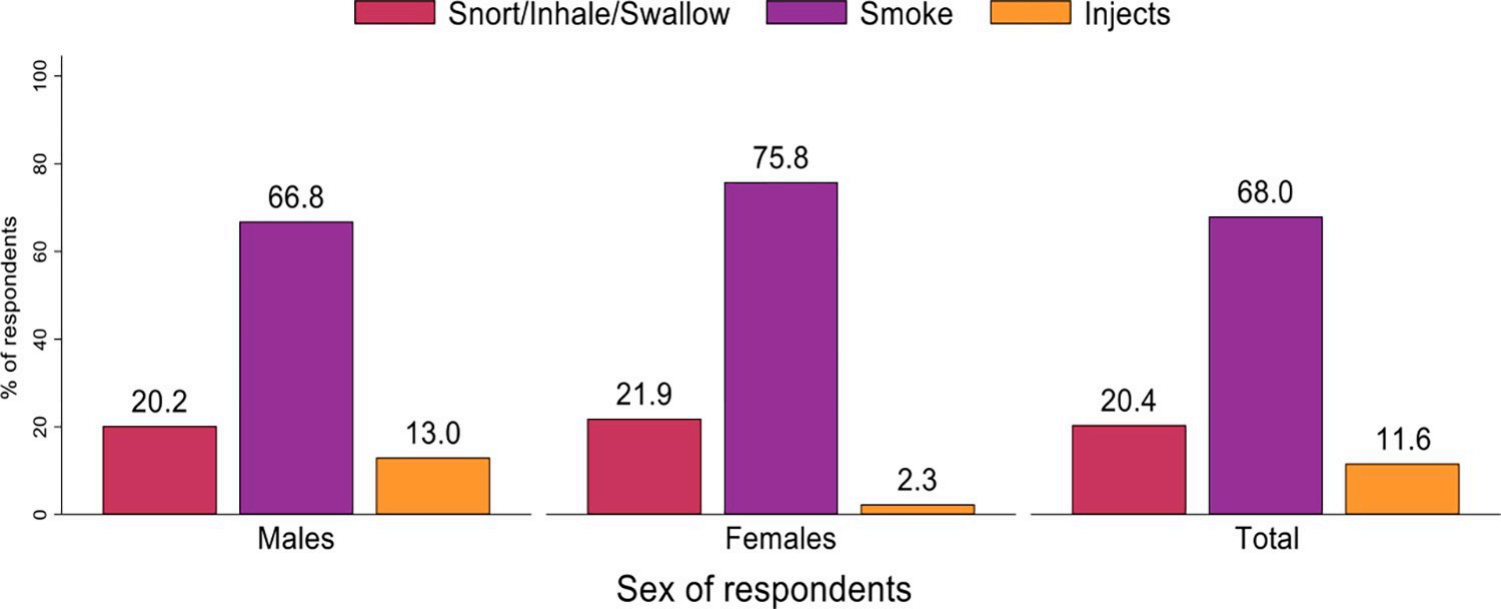
Methods of using drugs among respondents by sex.

*Methods of using drugs among respondents by sex*. Majority (68.0%) of the respondents consumed drugs through smoking, whilst 20.4% used drugs through snorting, inhaling or swallowing and 11.6% through injection, Fig 1.

**Fig 2:**
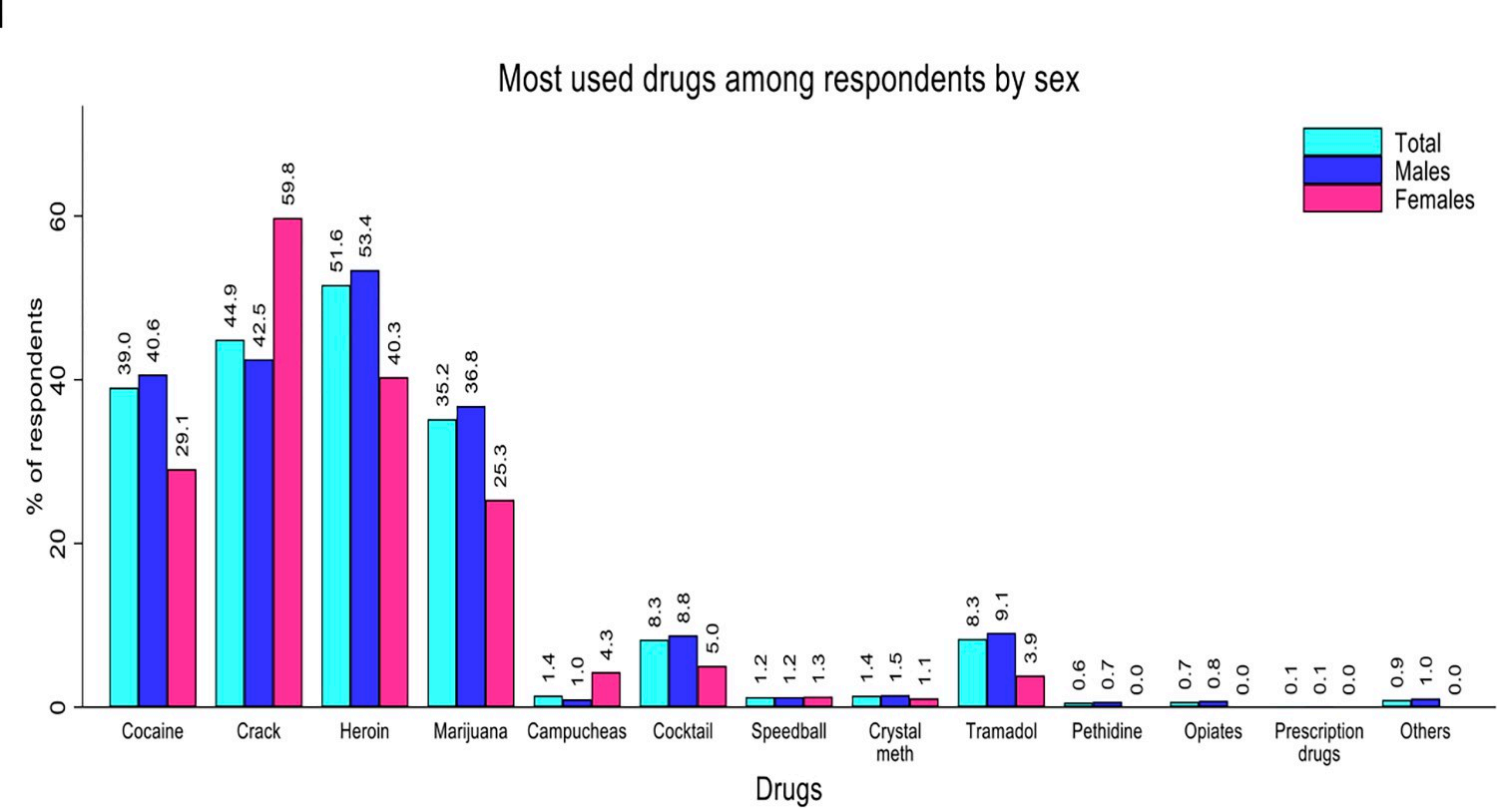
Most drugs used among respondents by sex.

*Most drugs used among respondents by sex*. The most used drugs were heroin (51.6%) followed by crack (44.9%). The distribution of drugs mostly used among respondents by sex is shown in Fig 2.

### Injection drug use

Most people got introduced to drug injection by friends and acquaintances (82.1%). About 12.4% of the PWID indicated that they mostly inject Crack. Among the 52 respondents who inject drugs, majority (51.1%) of them started injecting with cocaine. The most commonly injecting drugs among those who inject drugs was cocaine (55.2%). A quarter (25.8%) of the respondents had changed from the drugs the initially started injecting. Majority (52.7%) of the respondents changed to cocaine, Table 2.

**Table 2:** Characteristics of respondents who injects drugs by sex.

		Gender		
Factor	Total	Males	Females	
N	52 (%)	51 (%)	1 (%)	
**Age group at start of injecting drugs**				
** **<15 years	5.6	5.8	0.0	
** **15–19 years	23.9	21.9	100.0	
** **20–24 years	25.0	25.6	0.0	
** **25–29 years	16.6	17.1	0.0	
** **>30 years	28.9	29.7	0.0	
**Person who introduced injecting**				
** **A relative or family member	8.3	8.6	0.0	
** **A person you use drugs with	5.7	5.8	0.0	
** **A friend/an acquaintance	82.1	84.3	0.0	
** **A stranger	2.6	0.0	100.0	
** **Others	1.3	1.3	0.0	
**Drug injected at first time** ^**(M)**^				
*Cocaine*	51.1	52.4	0.0	
*Crack*	10.1	10.4	0.0	
*Heroin*	20.9	18.7	100.0	
*Marijuana*	0.8	0.9	0.0	
*Crystal meth / methamphetamine*	0.6	0.7	0.0	
*Tramadol*	8.9	9.2	0.0	
*Pethidine*	8.2	8.4	0.0	
**Ever changed from one injecting drug to the other**				
** **Yes	25.8	26.5	0.0	
** **No	69.9	69.1	100.0	
** **Non-response	4.3	4.4	0.0	
**What did it change to?**				
** **Cocaine	52.7	52.7	-	
** **Crack	16.9	16.9	-	
** **Heroin	10.9	10.9	-	
** **Crystal meth / methamphetamine	6.2	6.2	-	
** **Other	13.2	13.2	-	
**Last time injected drugs**				
** **Within the last 7 days	70.9	72.8	0.0	
** **More than 7 days to 1 month	6.1	6.2	0.0
** **More than 1 month up to 6 months	6.8	4.3	100.0
** **More than 6 up and to 12 months	6.5	6.7	0.0
** **More than 12 months	9.8	10.0	0.0
**Commonly injected drugs**			
** **Cocaine	55.2	56.8	0.0
** **Crack	12.4	12.8	0.0
** **Heroin	14.8	12.5	100.0
** **Crystal meth / methamphetamine	0.7	0.7	0.0
** **Tramadol	9.1	9.4	0.0
** **Pethidine	6.9	7.0	0.0
** **Other	0.9	0.9	0.0
**Number of times per day drug is injected, Median (IQR)**	2 (1, 4)	2 (1, 4)	2 (2, 2)
**Number of times per day drug is injected**			
** **1 time/day	27.6	28.4	0.0
** **2 times/day	25.2	23.1	100.0
** **3 times/day	17.5	18.0	0.0
** **4+ times/day	29.8	30.6	0.0
**Commonly use two or more drugs together**			
** **No	69.6	68.7	100.0
** **Yes	30.4	31.3	0.0

Almost all respondents (92%) pay to have access to needles and syringes. Frequency of injection was high with 70% injecting twice or more than twice a day. Majority (53%) of the people who inject drugs agreed that if you refuse to share or use needle or syringes with someone, they will consider it rude. Majority of people inject in ghetto/street (46%) and some alone (30%), Table 3.

**Table 3:** Needles and syringes use among PWID by sex.

		Gender	
Factor	Total	Males	Females
	n = 52	n = 51	n = 1
	%	%	%
**Source of needles/syringes in the last 6 months**			
** **Pharmacy/chemist/drug store/store/another store	57.4	56.1	100.0
** **Market place or street vendor	2.7	2.8	0.0
** **Pharmacy worker or drug vendor	20.7	21.3	0.0
** **Sex partner, friend, acquaintance, relative	5.3	5.4	0.0
** **Drug dealer or other drug users	9.0	9.3	0.0
** **Don’t know	4.9	5.0	0.0
**Sterile needles and syringes available when needed**			
** **Yes	80.6	80.0	100.0
** **No	10.8	11.1	0.0
** **Don’t know	8.7	8.9	0.0
**Pays for the needles**			
** **Yes	92.4	92.1	100.0
** **No	7.6	7.9	0.0
**Pay to be injected**			
** **Yes	35.5	36.5	0.0
** **No	64.5	63.5	100.0
**How often is a new sterile needle used in the last 6 months**			
** **Never	3.8	3.9	0.0
** **Rarely	9.0	9.3	0.0
** **Half of the time	12.0	9.6	100.0
** **Most of the time	14.7	15.1	0.0
** **Always	60.5	62.2	0.0
**Reasons for not using a new needle or syringe always**			
** **Not available	8.9	9.6	0.0
** **Difficult to find	26.9	28.9	0.0
** **Expensive	30.0	32.1	0.0
** **Peer pressured to share	20.9	22.4	0.0
** **I reuse my own needle	13.3	7.0	100.0
**When you inject, do you do it: alone, or with a friend, drugs dealer, assistant drug dealer**			
** **Alone	52.5	54.0	0.0
** **A friend	35.1	33.3	100.0
** **Drugs dealer	8.5	8.7	0.0
** **Assistant drug dealer	1.3	1.3	0.0
** **Don’t know	2.6	2.7	0.0
**In the last 6 months, how often did you use needles that someone else had already injected with**			
** **Never	67.4	69.2	0.0
** **Rarely	6.5	6.7	0.0
** **Half of the time	12.7	10.3	100.0
** **Most of the time	7.8	8.0	0.0
** **Always	1.3	1.3	0.0
** **Don’t know	4.3	4.5	0.0
**Shared needle with other person in the past 6 months**			
** **Not shared	52.2	53.8	0.0
** **Shared	31.6	29.5	100.0
** **Refused to answer	16.2	16.7	0.0
**Shared instrument with other person in the past 6 months**			
** **Not shared	50.0	51.5	0.0
** **Shared	50.0	48.5	100.0
**Venue or location drug is commonly injected**			
** **Own house	30.6	31.4	0.0
** **House of someone else	7.3	7.5	0.0
** **House of dealer	6.2	6.3	0.0
** **Abandoned building	4.3	4.4	0.0
** **Street (Ghetto)	46.2	44.7	100.0
** **Other places	5.5	5.7	0.0
**How often are needle/syringe reused before thrown out**			
** **Very Often	23.9	24.5	0.0
** **Often	15.0	12.6	100.0
** **Not so often	29.9	30.7	0.0
** **Never	31.3	32.2	0.0

#### Knowledge of respondents on HIV

**Fig 3:**
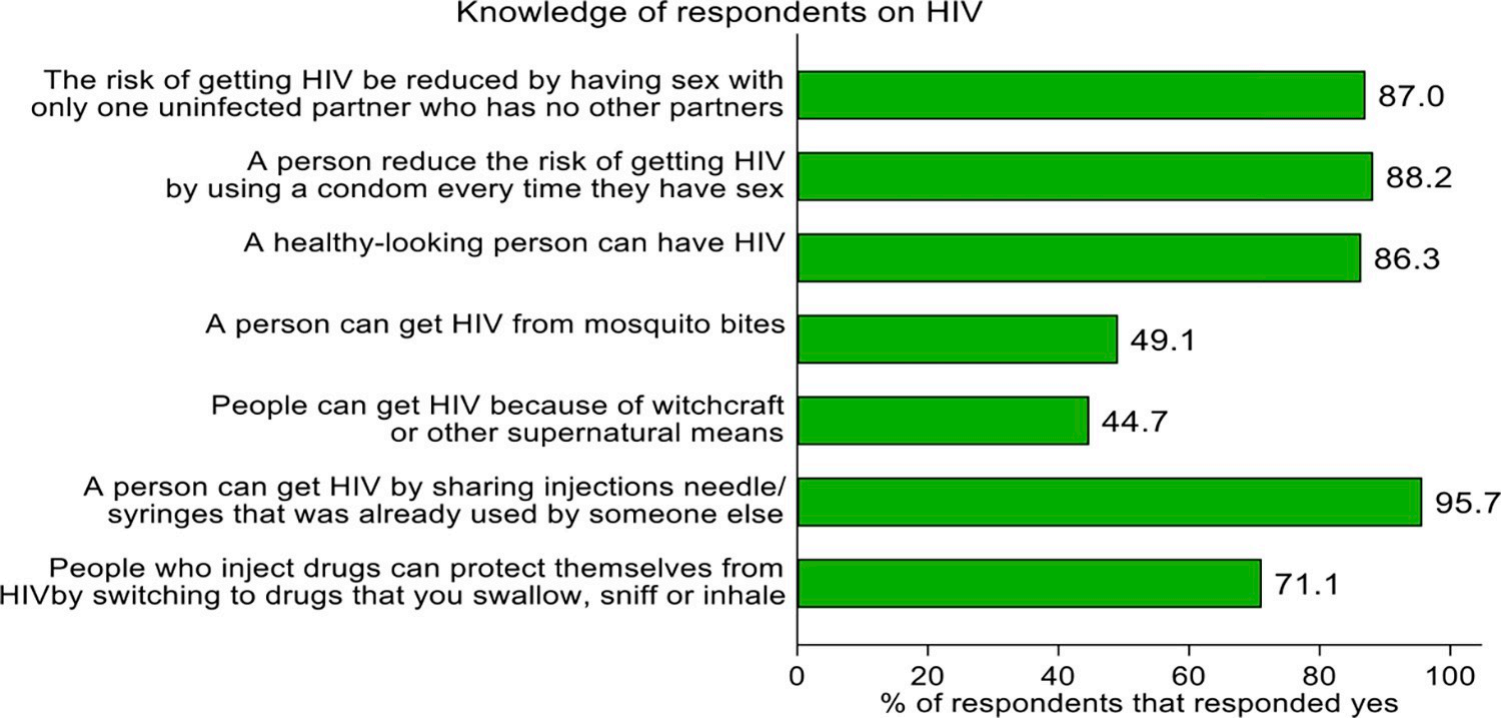
Knowledge of respondents on HIV by sex.

Majority (88.2%) of the respondents know that the risk of getting HIV is reduced by using condom every time they have sex. About 95.7% of the respondents also know that a person can get HIV by sharing injections needle/syringes that is already used by someone else. Most (71.1%) of the respondents also thought people who inject drugs can protect themselves from HIV by switching to drugs that a person can swallow or sniff or inhale, Fig 3.

#### Risky sexual behaviors among respondents

**Fig 4:**
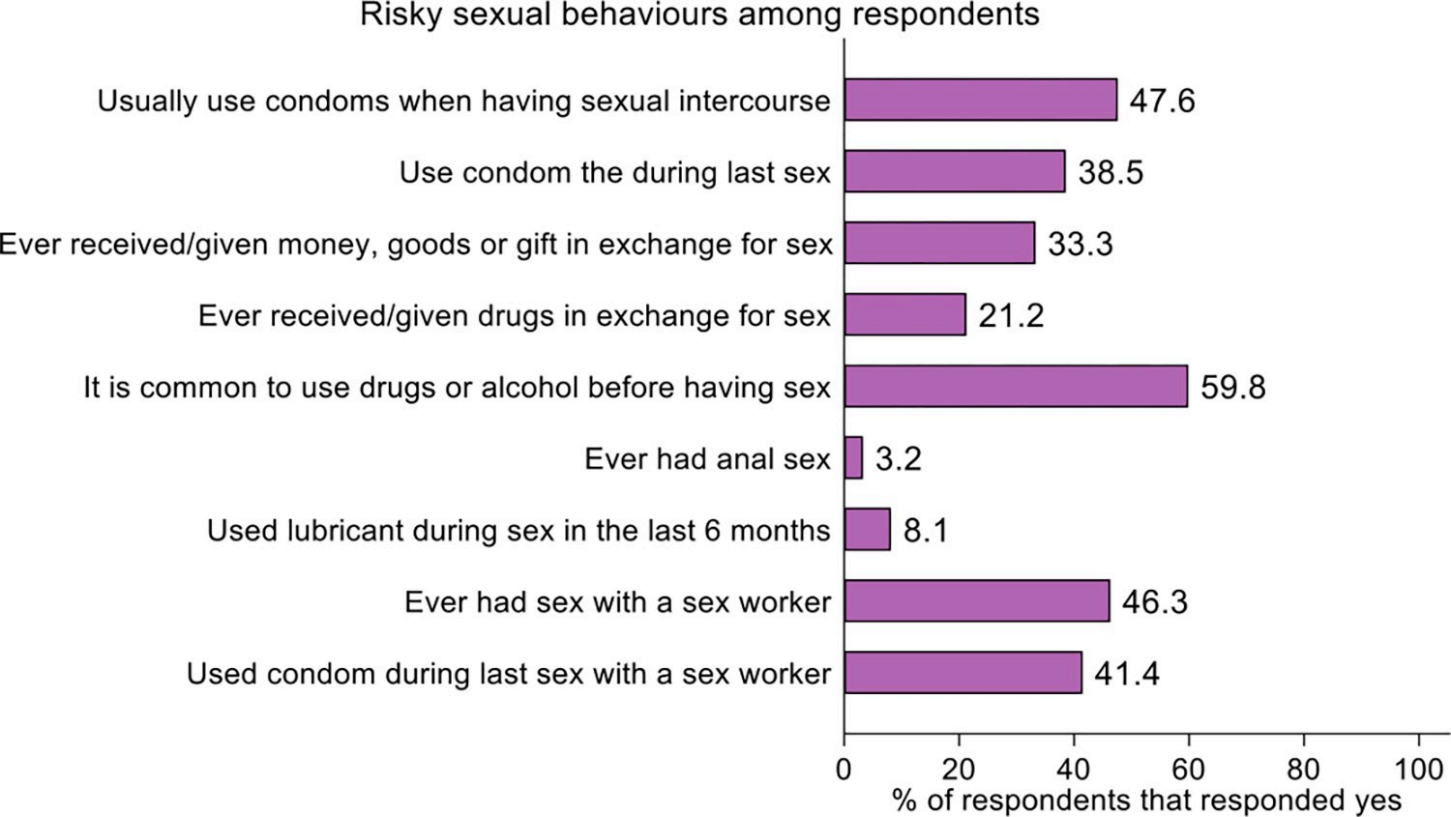
Risky sexual behavior among study respondents by sex.

A third (33.3%) of the respondents had ever received or given money, goods or gifts in exchange for sex whilst 21.2% had ever received or given drugs in exchange for sex. A few (3.2%) of the respondents had ever had anal sex. Less than half of the respondents have ever had sex with a sex worker (46.3%), Fig 4.

#### Sexually transmitted diseases and service uptake among respondents

A fifth (21.9%) of the respondents had some form of symptoms of sexually transmitted infections in the last 6 months. Abnormal discharge (9.1%), ulcer/sore from penis (6.7%), ulcer/sore from vagina (1.8%) and difficulty in urinating (13.4%) were some common STIs experienced. More than half (59.6%) of those who reported having symptoms of STIs sought treatment. Majority (63%) of the respondents have never had HIV test, one of the reasons being stigma by health care workers (96.5%). Only 51% of women are able to have access to family planning services when in need, Table 4.

**Table 4:** Sexually transmitted diseases and service uptake among respondents by sex.

				Gender	
			Total	Males	Females
N			323	278	45
Factor			%	%	%
**Had any symptoms of STIs in the last 6 month**					
** **No			78.1	78.0	78.6
** **Yes			21.9	22.0	21.4
**In the last 6months, which of the following symptoms of sexually transmitted infections have you had?**					
*Abnormal discharge*			9.1	7.8	17.0
*Ulcer/sore from your penis*			6.7	7.8	-
*Ulcer/sore from your vagina*			1.8	-	11.4
*Difficult urinating*			13.4	15.0	3.4
*Don’t know*			75.6	75.1	78.6
*Refuse to answer*			0.4	0.5	0.0
**Did you seek care/treatment because of these problems?**					
** **Yes			59.6	60.6	53.6
** **No			40.4	39.4	46.4
**If yes: Where did you go to get treatment?**					
** **Public clinic/hospital			35.3	34.7	40.2
** **Private clinic/hospital			7.4	8.5	0.0
** **Pharmacy			41.0	39.9	48.6
** **Other			16.2	16.9	11.2
**Has a peer educator or outreach worker ever talked to you about HIV?**					
** **Yes		46.9		46.0	52.4
** **No		50.8		52.0	43.5
** **Don’t know		0.4		0.5	0.0
** **Refuse to answer		1.8		1.5	4.2
**Have you ever tested for HIV?**					
Yes		35.6		31.0	64.2
No		63.0		67.3	35.8
Don’t know		0.8		0.9	0.0
Refuse to answer		0.7		0.8	0.0
**Reasons for not testing for HIV** ^**(M)**^					
*I feel I am not at risk for HIV*		53.3		55.0	32.9
*Fear of positive result*		89.7		89.4	92.8
*No money to get tested*		81.6		81.4	83.8
*No time to get tested*		73.0		71.1	94.6
*Stigma by health care workers*		96.5		96.2	100.0
*Others*		89.1		88.9	92.2
**What was the result of your last test?**					
** **Positive		1.8		0.0	7.2
** **Negative		86.4		87.2	84.1
** **Did not receive result		0.3		0.4	0.0
** **Don’t know		11.4		12.4	8.7
**(Females only) Have you ever been pregnant?**					
** **Yes		78.9		-	78.9
** **No		13.0		-	13.0
** **Refuse to answer		8.1		-	8.1
**(Females only) Are you able to get family planning services if you want them? That is to either prevent pregnancy or to limit number of children**					
** **Yes		50.9		-	50.9
** **No		44.0		-	44.0
Refuse to answer	5.1			-	5.1

**Fig 5:**
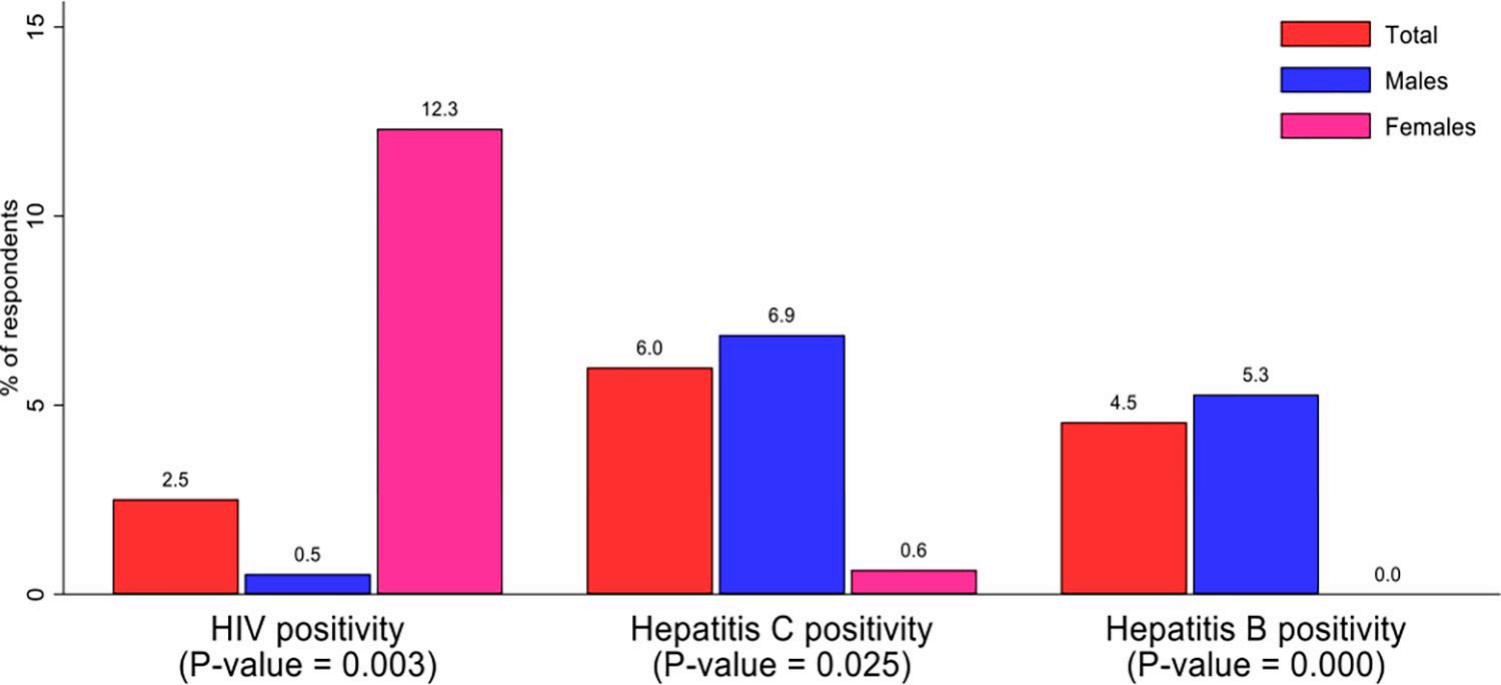
Prevalence of HIV, hepatitis C and hepatitis B infection among respondents by sex.

**Fig 6:**
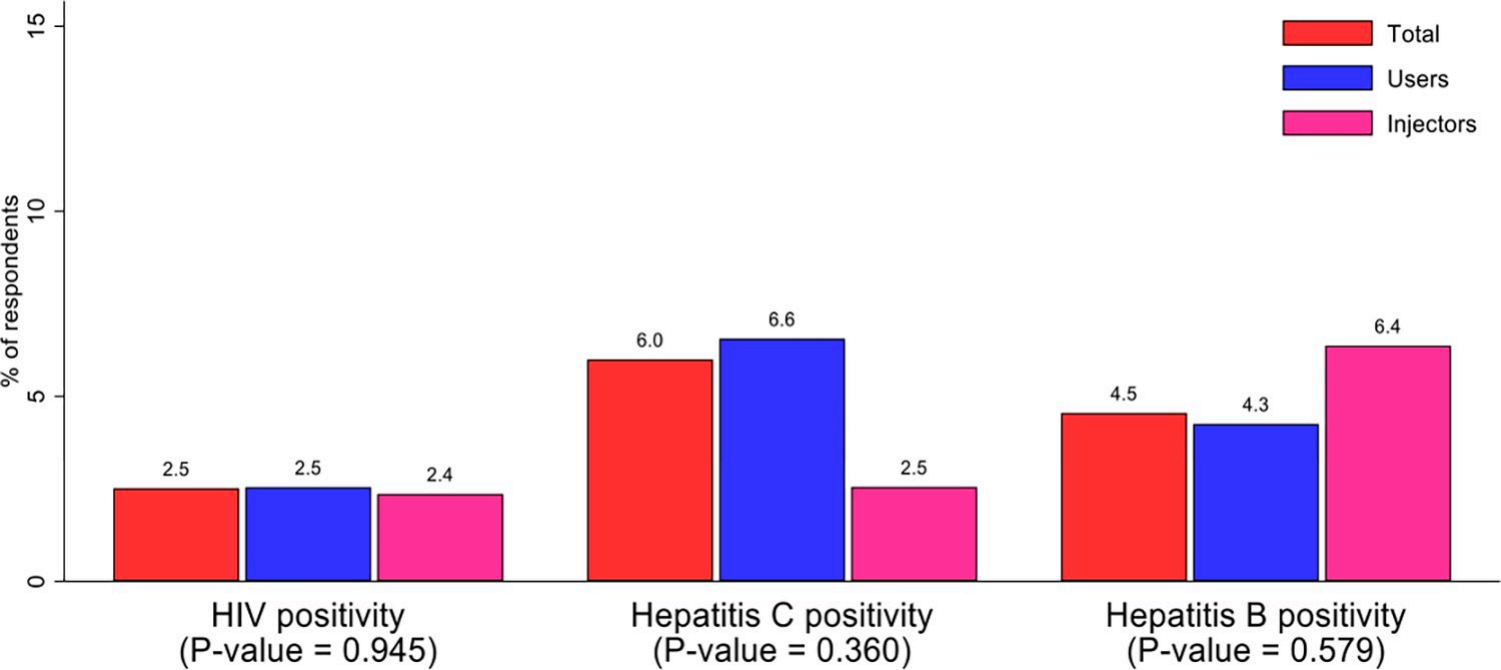
Prevalence of HIV, hepatitis C and hepatitis B infection among respondents by drug use and injection type.

Prevalence of HIV among the respondents was 2.5%. HIV prevalence was higher among the females (12.5%). The prevalence of hepatitis C was 6.0% and high among males (6.9%). Hepatitis B prevalence was 4.5% among the respondents. There was statistically significant difference in the HIV prevalence by sex (p-value = 0.003). There was also a statistically significant difference in Hepatitis B and C prevalence by sex, Fig 5. HIV prevalence was 2.5% among PWUD and 2.4% among PWID. Hepatitis B was observed to be higher in PWID and hepatitis C in PWUD, Fig 6.

## Discussion

As in many West African countries, Ghana has become a transit country for heroin and cocaine over the past decades, with previous reports of local demand for those drugs growing over the period [12]. People who inject or use drugs are among the key populations most at risk to acquire or transmit HIV. These group of people are 22 times more at risk of HIV compared with the general population [13]. This risk of infection arises particularly from sharing needles and injection equipment but is reinforced through criminalization, marginalization, and poverty. Yet they are also among those with the least access to HIV prevention, care and treatment services because their drug use is often stigmatized and criminalized [13]. This important key population group of people remain under-served in Ghana. It is important not only to recognize their vulnerabilities but also to take concrete steps to provide appropriate services that address their needs. This research presents findings on a rapid situational assessment of drug use and injection in four regions (Greater Accra, Ashanti, Western and Northern) of Ghana.

Overall, there were more males interviewed with a median age of 37 years. The median age at which they started using and injecting drugs was 20 and 22 years, respectively. Studies from elsewhere, show that drug users were younger (32 years), there were in general, older than what was found in this study [14–16]. People within the lower socio-economic status were the majority of those who use and inject drugs. Though people of higher socio-economic status also use drugs, they use it in their homes and do not go so often to the ghettos making them very hard to reach. It is reported in literature that people who are classified as substance addicts are not all necessarily disadvantaged or poverty-stricken but rather affluent young adults and adolescents of higher socioeconomic status [17, 18].

There was a higher percentage (68%) of drug consumption through smoking with only 11.6% injecting. Confirmed with a similar finding, most injecting drug users smoke their drugs in comparison to injecting them [19]. A study reports a little over 80% of injecting drug users smoking their drugs as compared to those who actively injected theirs (40%) [20]. Though majority of the respondents started using marijuana as a drug followed by heroin, about 29.1% switched to cocaine over time. A recent study confirms marijuana as the mostly used drug by new drug users with cocaine as part of the frequently used drugs during initiation [21]. Cocaine, heroin and tramadol were mostly consumed by the males. The crack substance was mostly consumed by Females. Contrary to a previous study, males mostly used alcohol, marijuana and anabolic androgenic steroids (AAS). Women on the other hand used amphetamines, tranquilizers and opiate analgesics [22].

Polydrug use was common among the population, with about 64.7% saying they commonly use two or more drugs together while one-third mixed alcohol with other drugs. Literature reports of significant polydrug use among a great proportion of drug users. These drugs are either consumed at the same time or at different intervals, however, they report the consumption of more than only one drug by injecting drug users. Alcohol was also well utilized by some of these polydrug users [23–26]. The assessment showed that drug injection was relatively uncommon among females. A recent study in congruence with this finding showed that about 8% of study participants who injected drugs were females, suggesting the significantly higher practice of drug injection among males than females [27].

Knowledge about HIV transmission was generally modest. Some of the injecting drug users believe that HIV can be transmitted through witchcraft and mosquito bites. Other available studies recorded similar trends where quite a high number of respondents believe that through mosquito bites, people can be infected with HIV; noteworthy are also participants who are of the view that through supernatural means such as witchcraft, one can acquire the HIV disease [28–30]. Condom use was observed to be low among drug users in the study. This was in agreement with an existing study where PWID were inconsistent condom users especially when they had casual sex [31]. The assessment illustrates gaps in HIV prevention which requires attention and poses a risk for transmission of HIV and hepatitis within the cluster of PWID.

HIV prevalence was 2.5%, higher than the national average, but no significant difference existed between PWID and non-PWID, this could imply that the risk of transmitting HIV for PWID in this study is relatively low. A survey conducted in Mozambique among PWID reported a very high prevalence of HIV (50.1%) in Maputo and (19.9%) in Nampula. These figures are similar to those reported in South Africa (21%), Mauritius (45.5%) and Kenya (42%) but markedly different from the prevalence reported in this study and those reported in Nigeria (3.1%), Madagascar (4.8%) and Seychelles (3.8%) [32–34].

HIV prevalence among women was disproportionately very high (12.5%). This is partly because women who use drugs have overlapping vulnerabilities such as risky behaviors, including engagement in sex work. As a previous study stated, HIV has become a ‘feminine’ pandemic due to its high occurrence in the female population as compared to the male gender [35–37]. Despite this high prevalence of HIV among women, we found no tailored services for women. As witnessed in other contexts, HIV prevalence can rapidly increase with increased injection practices and other risky behaviours [38–41].

The overall prevalence of HCV in this study was 6.0% while that of HBV was 4.5%. Hepatitis B and C were more prevalent among males in the study as opposed to females. Literature confirms this gender disparity in viral hepatitis to the disadvantage of men. This is due to the different properties of the male and female sex hormones, leading to varying effects when both are exposed to these viruses [36, 42]. Global estimates of HBV among PWID was 9.1% which constitutes about 1.4 million people while that of HCV was 52.3% constituting about 8 million people but in sub-Saharan Africa, the prevalence of HBV was 3.7%. and that of HCV was 21.8%. Most countries in sub-Saharan Africa had similar HBV prevalence estimates as found in this study, Kenya (5.4%), Madagascar (5.0%), Mauritius (6.1%), and Nigeria (6.7%). For HCV, the prevalence among sub-Saharan Africa countries varied with the highest in Mauritius (97.1%), Mozambique (67.1%), Seychelles (42.0%) and Ghana (40.1%) [9, 32, 41].

In this study, women, have reported not being able to have access to family planning services. Additionally, one-fifth of these women who reported symptoms of STI in the last 6 months were not able to access treatment services. As evidence from other contexts shows, women’s drug use remains hidden as they may be more concerned about being exposed. Furthermore, women who use drugs face increased stigma, discrimination and rejection from community and health care professionals and are subject to high rates of sexual violence from law enforcement officials. This discourages accessing health care and/or disclosure of their drug use when using health services due to fear of prosecution, harassment and assault. Many women rely on their (often male) partners to provide injecting equipment and to assist with injecting, and they are less likely to access health care services. Women who use drugs are also noted to be at an elevated risk of gender-based violence, HIV infection, sexually transmitted infections and unplanned pregnancies and have limited access to sexual and reproductive health services. This can have negative effects on pregnant women and mothers. Drug use during pregnancy is criminalized in some jurisdictions, and women could face prosecution for using drugs [43–48].

Half of our respondents have not been reached through outreach, and 63% have never been tested for HIV. This is significantly less than HIV testing among female sex workers (75%) in the integrated biobehavioral surveillance survey in 2020 in Ghana. Reasons for not testing included feeling that they are not at risk for HIV, fear of positive test results, having no money to get tested, having no time to get tested, and fear of stigmatization from health workers. About three-quarter of the respondents have never tested for HBV and 86% never tested for HCV. A report by Center for Disease Control and Prevention (CDC), recommends the need for routine hepatitis B and C testing for people who continue to engage in high-risk behaviors [49]. Human immunodeficiency virus (HIV) and Hepatitis prevention, including expansion of testing and screening services among this population, will help mitigate the transmission risk. Injection drug use is a key primary factor in the transmission of blood-fluid-borne pathogens with the infection of HIV virus exacerbated by tuberculosis infection [16]. Studies have shown that injecting drug users have an increased risk of sharing injection paraphernalia and engaging in high unprotected sexual activities (lack of condom use, multiple sexual partners) and all these increase the risk of spreading HIV, HBV and HCV [50–52]. As stated in Guure et al., the prevalence of HIV (2.5%), HBV (4.1%) and HCV (6.7) are not very alarming among this key population in Ghana. Interventions such as screening, early intervention and the scale-up of therapeutic options such as antiretroviral therapy (ART), drug treatment and long-term HIV, HBV and HCV care for injecting drug users are essential for effective control of co-infections [51].

## Recommendations

- Further research is required to assess whether the implementation of the comprehensive package of interventions for the prevention, treatment and care of HIV is being carried out among people who use and inject drugs.
- More research is needed to determine whether ART, Hep C and Hep B treatment and vaccinations are provided to those infected in line with WHO recommendations.
- Further research work is needed to establish the magnitude of stigma and discrimination against PWID and PWUD by health care workers, police and law enforcement agents, and measures taken to mitigate them.

### Limitations

Some of the respondents did not know the names of some of the drugs and were only familiar with the street names. However, the same street name could mean different drugs to different users in various locations. Although names per region were cross-checked, the study could not verify if the street names mentioned by respondents refer to the appropriate drug names. Additionally, though the sample size for the study was adequate for national level analysis, it was not powered for subnational analysis and comparisons. Furthermore, the frequencies for HIV, HBC and HCV were not adequate enough to allow for the establishment of factors associated with each and so no regression analysis was carried out. The assessment was done in four regions of Ghana, and although these were the regions with higher reported drug use, the findings in this report do not necessarily describe the characteristics and situation for all PWUD/ PWID in Ghana. Lastly, due to the high stigma among PWUD/ PWID, individuals who are members of this group may not have revealed behaviors and risks resulting in self-reporting response bias.

## Conclusion

HIV prevalence among PWUD/PWID is higher than the general population rate although lower than other key populations (female sex workers, men who have sex with men and transgender). Women have higher prevalence and are at an increased risk, with overlapping vulnerabilities of drug use/injection and engagement in sex work. Polydrug use is widely common in Ghana, with community of PWUD engaging in risky injection and sexual behaviors that put them at high risk of contracting HIV, STIs and Hepatitis. Key HIV/AIDS services–such as screening and treatment for HIV, and Hepatitis or educational outreach related to HIV/AIDS–are not reaching PWUD/PWID in any sustained manner. Harm reduction programs are nonexistent, and the quality of drug treatment services are largely unavailable and/or too expensive. Ghana has a window of opportunity to launch interventions that will help reduce the spread of HIV and Hepatitis among PWUD/PWID. However, authorities must be aware that opportunity must be timely, as HIV and other blood-borne infections spread by sharing of injecting equipment more quickly than with sexual transmission of the virus.

## Supporting information

S1 Data (CSV)
